# No Room for Error: Empiric Treatment for Fulminant Pneumonia

**DOI:** 10.5811/cpcem.2017.1.33213

**Published:** 2017-03-13

**Authors:** Matthew E. Prekker, Stephen W. Smith

**Affiliations:** * Hennepin County Medical Center, Department of Emergency Medicine, Minneapolis, Minnesota; † Hennepin County Medical Center, Department of Medicine, Division of Pulmonary and Critical Care Medicine, Minneapolis, Minnesota; ‡ University of Minnesota Medical School, Minneapolis, Minnesota

## Abstract

Early antibiotic administration is critical in cases of sepsis and severe community-acquired pneumonia, which is frequently due to *Streptococcus pneumoniae*, *Staphylococcus aureus*, *Legionella* species, or influenza. We describe the case of a 29-year-old previously healthy man who presented to an urban emergency department (ED) in the North Central U.S. with fever, hip pain, severe hypoxemia, and diffuse pulmonary infiltrates. He was intubated and received piperacillin/tazobactam, levofloxacin, vancomycin, and oseltamivir; given his fulminant presentation and predicted high mortality, doxycycline, methylprednisolone, and amphotericin B were also administered empirically in the ED. A respiratory culture eventually grew *Blastomyces dermatitidis*, and the patient survived. Severe acute respiratory distress syndrome due to fulminant pneumonitis carries a high mortality. Faced with this scenario and no room for error, it is important that the emergency physician cover for all possible pathogens, including zoonotic bacteria and endemic fungi.

## INTRODUCTION

Severe community-acquired pneumonia (CAP) is a frequent cause of sepsis and the acute respiratory distress syndrome (ARDS).^1^ However, a fulminant presentation of these syndromes in an otherwise healthy patient in the emergency department (ED) is uncommon.^2^ Severe CAP is usually due to one of a familiar list of bacterial and viral pathogens (*Streptococcus pneumoniae*, *Staphylococcus aureus*, *Legionella* sp., and influenza A&B viruses), and effective antimicrobial therapy in the first hour after presentation to the ED improves the likelihood of a good outcome.^3^,^4^ Furthermore, seasoned emergency physicians (EP) understand the importance of matching the intensity and timeliness of their interventions (including empiric antimicrobial therapy) to the severity and tempo of the patient’s illness; in general, a fulminant presentation of sepsis and pneumonia in a young adult demands rapid, definitive intervention to preserve life. Surprisingly, existing literature and guidelines give little guidance to the EP regarding an approach to empiric antimicrobial therapy for fulminant, immediately life-threatening pneumonia.

## CASE REPORT

A 29-year-old previously healthy male presented with high fever, tachycardia, hypoxia and bilateral, dense pulmonary infiltrates. The patient had no prior medical history. He was from a farming community in rural Minnesota, and had been outside working in the month of May. He had been feeling well until five days prior when he developed severe right-sided hip and back pain and sought medical attention, for which he received an unspecified spinal “injection.” Two days later (three days prior to ED presentation) he developed a cough and was prescribed azithromycin. During the following two days his cough became worse and, on the morning of presentation, he awoke with dyspnea. He presented to a clinic and was found to be hypoxic and febrile, and was sent to the local community ED. On arrival, the patient was in severe distress, with a temperature of 40 degrees C, tachycardia, and an oxygen saturation of 45% on room air. He was intubated and placed on mechanical ventilation. A chest radiograph showed diffuse pulmonary infiltrates, and he was administered 2 grams (g) of ceftriaxone and prepared for transfer to a tertiary care center. Prior to departure, on recommendation of the accepting facility, piperacillin/tazobactam 4.5 g, vancomycin 2 g (approximately 22 milligrams per kilogram [mg/kg]), as well as methylprednisolone 125 mg were given intravenously.

**Image 1:**
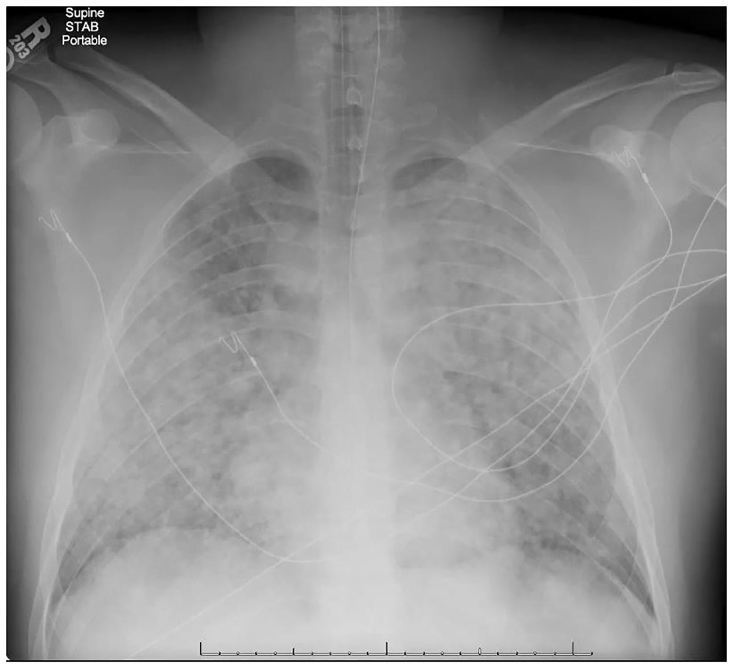
Anteroposterior chest radiograph taken in the emergency department showing bilateral, diffuse pulmonary opacities consistent with the acute respiratory distress syndrome.

On arrival at the tertiary care center, the patient was a well-developed, well-nourished young male who was intubated, sedated, and pharmacologically paralyzed. Pulse was 160 beats per minute, blood pressure 200/100 mm Hg with bounding pulses, temperature 39 degrees C, and an oxygen saturation of 88% on 100% inspired oxygen fraction (F_I_O2). Breath sounds were equal and very coarse. A chest radiograph showed bilateral pulmonary infiltrates (Image 1), and an electrocardiogram showed only sinus tachycardia.

Point-of-care cardiac ultrasound showed hyperdynamic function and an ultrasonic cardiac output monitor (USCOM®) calculated a cardiac output of 19 liters/minute, with stroke volume of 120 milliliters and systemic vascular resistance of 550 dyn·s/cm,^5^ Using point-of-care ultrasonography, we found that the inferior vena cava was neither distended nor collapsed and the painful hip showed no excess joint fluid.

Electrolytes were normal. Hemoglobin was 14.2 g/dl. Leukocyte count was 46,300 cells/mm^3^ with 68% neutrophils and 23% band forms. Arterial blood gas returned with a pH of 7.21, pCO2 of 48 mm Hg, pO2 of 74 mm Hg, and bicarbonate of 18 mEq/L. The lactate was 3.2 mEq/L, albumin 2.6 g/dL. The international normalized ratio was 1.6. Human immunodeficiency virus antibody was nonreactive. A sputum sample obtained by tracheal aspirate showed a negative gram stain, and sputum and blood cultures were sent for analysis.

Several liters of normal saline were administered in bolus fashion without change in vital signs. A positive end-expiratory pressure (PEEP) of 10 cm H_2_O was administered, with improvement in pulse oximetry to 91%. With the assessment that this patient required immediate, effective empirical antimicrobial therapy for a fulminant pneumonia of unknown etiology, the patient received levofloxacin 750 mg IV, oseltamivir 150 mg enterally, doxycycline 100 mg IV, and liposomal amphotericin B 300 mg IV (approximately 3 mg/kg) in the ED.

**Image 2:**
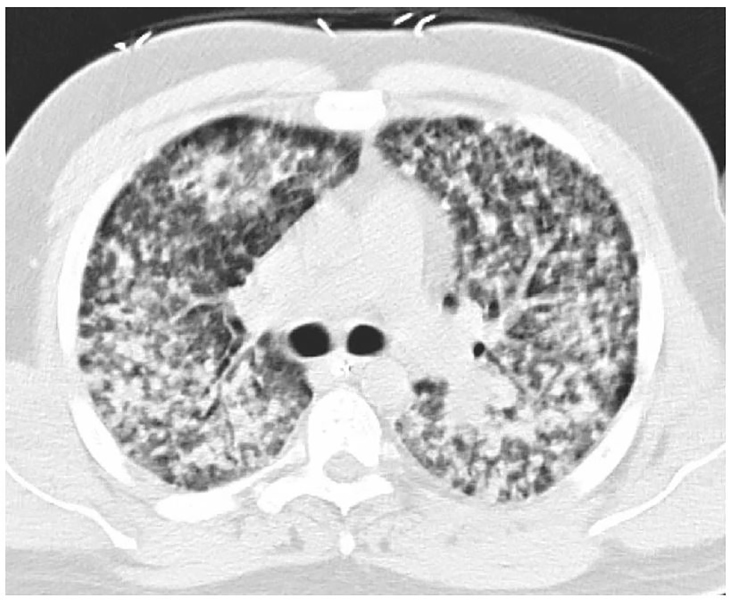
Axial image at the level of the mainstem bronchi taken from a computed tomography of the chest obtained in the emergency department, showing diffuse, nodular opacities in both lungs.

A computed tomography of the chest and abdomen was done to better define the pattern of pneumonitis and to further investigate the severe back and hip pain. It only showed pathology in the chest (Image 2).

The patient was admitted to the medical intensive care unit (ICU). Broad spectrum antimicrobials were continued. The next day, broad-based budding yeast were seen on a silver stain of bronchoalveolar lavage (BAL) fluid, and subsequently fungal cultures of both sputum and BAL samples grew *Blastomyces dermatitidis*. Liposomal amphotericin B was continued and all other antimicrobials were discontinued. After one week of amphotericin and supportive care, the patient began to recover. Eight months later, he was being treated with itraconazole (planned 12-month course); he had a normal chest radiograph, but still had some airflow limitation and dyspnea on exertion.

## DISCUSSION

This case is, to our knowledge, the first report of ARDS due to fulminant pulmonary blastomycosis in the emergency medicine literature. While this entity is uncommon, it has been described in ICU patients. Of note, an experienced EP, faced with a young patient with undifferentiated, near-fatal CAP, recognized the importance of definitive antimicrobial therapy and prescribed broad spectrum antibiotics including doxycycline (for zoonotic bacterial pathogens), as well as empiric antiviral and antifungal therapy. This patient’s blastomycosis was covered empirically (and as it turns out, definitively) with amphotericin from the time of his arrival in the ED. This report addresses one potential approach to fulminant pneumonitis from an unknown pathogen, which represents an important gap in existing guidelines on early antimicrobial therapy.

There is ample evidence that a delay in definitive antibiotic therapy negatively affects outcomes in severe bacterial infection. This delay is most often due to unrecognized infection, failure to initiate antibiotic therapy in the ED, or failure to anticipate antimicrobial resistance. In fact, among hypotensive ICU patients with septic shock (nearly 40% of whom had pneumonia), each hour delay in effective antimicrobial therapy after the first hour was associated with an average decrease in survival of 8%.^3^ The Surviving Sepsis Campaign, while not addressing fulminant pneumonia specifically, does recommend that empiric antimicrobial therapy include one or more drugs that have activity and adequate tissue penetration against “all likely pathogens,” including viruses and fungi.^5^ Professional society guidelines on the management of critically ill patients with severe CAP highlight the need to cover empirically for resistant organisms including *Pseudomonas aeruginosa* and community-acquired methicillin-resistant *Staphylococcus aureus* (CA-MRSA).^6^ In our experience, patients with septic shock rarely receive antifungal or antiviral therapy. Besides knowledge of non-bacterial pathogens endemic to a certain geographic area (e.g. *Coccidioides* spp. and hantavirus in the southwestern U.S.), these guidelines are of limited utility to an EP caring for an intubated patient because treatment is initiated before a detailed travel and exposure history can be obtained.

A diverse list of pathogens can cause fulminant pneumonia and ARDS in an immunocompetent host. In fact, there is evidence that multilobar lung involvement is independently associated with a twofold increased likelihood of treatment failure in CAP.^7^,^8^ Usual pathogens include standard or atypical bacteria, such as *Streptococcus pneumoniae*, *Haemophilus influenzae*, *Staphylococcus aureus*, Group A Streptococcus, *Legionella* spp., or aerobic gram-negative bacteria, including *Pseudomonas aeruginosa*. Massive aspiration can lead to a polymicrobial pneumonia that often includes anaerobes, or in the case of freshwater aspiration, infection with *Aeromonas hydrophila*.^9^ In our case, antibacterial coverage included piperacillin-tazobactam, levofloxacin, and vancomycin. Endemic fungal infections, such as blastomycosis, histoplasmosis, or coccidioidomycosis, have also been associated with fulminant pneumonia; thus, we gave amphotericin. One must also consider viruses such as influenza A and B (for which we gave oseltamivir), but also varicella zoster virus^10^ or herpes simplex,^11^ and thus it may be prudent to administer acyclovir in the appropriate setting. Respiratory viruses that are prevalent, detectable on polymerase chain reaction-based assays but without specific treatment include respiratory syncytial virus, parainfluenza virus, rhinovirus, adenovirus, human metapneumovirus, multiple coronaviruses (the etiologic agents of severe acute respiratory syndrome [SARS] and of Middle East respiratory syndrome [MERS]), and hantaviruses (responsible for the hantavirus pulmonary syndrome [HPS]). Fulminant pneumonia can also be caused by rare zoonotic bacteria often recognized in the U.S. for their potential as biological weapons, namely *Bacillus anthracis* (anthrax), *Francisella tularensis* (tularemia), and *Yersinia pestis* (pneumonic plague), for which we gave doxycycline. Lastly, fulminant pneumonitis can be due to non-infectious vasculitic or idiopathic disorders, which are typically corticosteroid-responsive, and methylprednisolone was given in this case.

Blastomycosis can be asymptomatic or mimic bacterial pneumonia following the inhalation of aerosolized spores from the *Blastomyces dermatitidis* mold living in moist soil. This endemic fungus is found most commonly surrounding the Great Lakes and the St. Lawrence, Ohio, and Mississippi Rivers.^12^ In states where blastomycosis is a reportable disease (Arkansas, Louisiana, Michigan, Minnesota, and Wisconsin), it is relatively rare; annual incidence rates vary from 1–2 cases per 100,000 population to as high as 10–40 cases per 100,000 population in several northern Wisconsin counties.^13^,^14^ Fulminant pneumonia leading to ARDS and respiratory failure occurs in a minority of cases, with mortality of 50–89%. (Contemporary mortality may be lower in the era of extracorporeal membrane oxygenation support).^15^,^16^ Extrapulmonary blastomycosis can occur by hematogenous spread of yeast to the skin, bones, and joints. Delay in diagnosis is relatively common; clinicians even in endemic areas often fail to consider blastomycosis in the initial differential for severe CAP. The diagnosis is made by isolating the organism in culture. Rapid evidence of blastomycosis is often obtained by visualizing the broad-based budding yeast forms in a sputum smear or potassium hydroxide prep. In severe infections, *Blastomyces* can cross-react with the urine *Histoplasma* antigen assay yielding a positive result before the final culture is available, as occurred in our case. Treatment of severe pulmonary or disseminated blastomycosis is with liposomal amphotericin B for 1–2 weeks until clinical improvement is noted, then daily itraconazole for at least one year.^17^

## CONCLUSION

Truly fulminant cases of community-acquired pneumonia in ED patients are rare but life-threatening, as in this case of acute pulmonary blastomycosis presenting as ARDS. In such patients with no room for error (where a delay in definitive antimicrobial therapy may be disastrous), consider extending empiric therapy to cover endemic fungi (amphotericin B) and zoonotic infections such as pneumonic plague or tularemia (doxycycline or an aminoglycoside).
